# Knowledge, practices, and challenges in primary care management of dizziness and vertigo in Saudi Arabia

**DOI:** 10.3389/fneur.2026.1793772

**Published:** 2026-03-13

**Authors:** Reem Elbeltagy, Aljawharah Algarni, Arwa Altamimi, Shadan Alotaibi, Rana Alanazi, Lama Alenazi, Norah Alfuhid, Rania Alkahtani, Sultan Alkahtani

**Affiliations:** 1 Department of Health Communication Sciences, College of Health and Rehabilitation Sciences, Princess Nourah Bint Abdulrahman University, Riyadh, Saudi Arabia; 2 Security Forces Hospital Program, Riyadh, Saudi Arabia

**Keywords:** diagnosis, dizziness, primary care, Saudi Arabia, treatment, vertigo

## Abstract

**Background:**

Dizziness and vertigo are common and challenging complaints in primary care, yet often remain underdiagnosed or mismanaged. This study aimed to assess primary care physicians’ (PCPs) knowledge, diagnostic practices, satisfaction, and perceived needs related to the management of dizziness and vertigo in Saudi Arabia.

**Methods:**

A cross-sectional survey was conducted among 186 PCPs across Saudi Arabia using an online questionnaire. The survey assessed sociodemographic characteristics, diagnostic and treatment practices, knowledge (7-item Likert scale), satisfaction (single-item scale), and suggested areas for improvement.

**Results:**

Most respondents were under 30 years old (53.2%) and had less than 5 years of clinical experience (57.5%). While 54.3% reported seeing more than five dizziness cases monthly, only 39.8% allocated additional consultation time. Basic diagnostic tools were variably available; 13.4% reported having none. The mean knowledge score was 20.9 (SD ± 3.99), with 16.1% showing poor knowledge. Satisfaction with dizziness management was modest (mean score 3.12 ± 0.98), and only 31.2% reported being satisfied. Knowledge positively correlated with satisfaction (*r* = 0.495, *p* < 0.001), years of experience, time per patient, and dizziness case volume.

**Conclusion:**

Despite moderate knowledge levels, PCPs face significant limitations in tools, training, and systemic support for managing dizziness. Enhancing PCP education, expanding access to basic diagnostic resources, and strengthening collaboration with vestibular specialists, such as audiologists, could improve diagnostic accuracy, physician satisfaction, and patient outcomes in Saudi primary care.

## 1 Introduction

Vestibular disorders, encompassing a broad spectrum of disorienting sensations like dizziness, vertigo (spinning sensation), presyncope (lightheadedness), and disequilibrium (imbalance), are a prevalent concern globally (1). According to a large population-based study, dizziness (including vertigo) affects about 15% to over 20% of adults yearly (2). Vestibular vertigo specifically accounts for nearly one-quarter of all dizziness complaints, with a 12-month prevalence of 5% and an annual incidence of 1.4% (2). Its prevalence rises with age and is about two to three times higher in women than in men (2).

These symptoms can be profoundly debilitating and have a significant impact on individuals’ daily functioning and quality of life (3). Patients often report difficulty performing routine tasks, reduced mobility, and decreased participation in social or occupational roles (4, 5). Dizziness is also among the leading symptoms prompting emergency room visits and contributes substantially to increased healthcare utilization, especially among the elderly (6). Furthermore, mental health conditions such as anxiety, depression, and phobias can exacerbate dizziness symptoms, contributing to avoidance behaviors, functional disability, and increased healthcare utilization (7, 8). In older adults, vertigo and dizziness are also associated with an elevated risk of falls and fall-related injuries, posing a major public health concern (9).

Vertigo and dizziness are among the most common complaints in primary care settings, particularly among women and older adults (10). However, these symptoms are frequently left undiagnosed or misdiagnosed by primary care physicians (PCPs), often due to their episodic nature, non-specific presentations, and the complexity of vestibular disorders (11). Importantly, differentiating between benign peripheral vestibular conditions and potentially life-threatening central causes remains a diagnostic challenge in primary care (12). Failure to identify red flags or perform appropriate bedside testing may result in delayed diagnosis and poor outcomes. This diagnostic uncertainty underlines the need for evidence-based clinical guidelines and decision-support tools tailored for use in primary care.

A systematic review of dizziness symptoms in primary care highlighted the urgent need for practical diagnostic approaches and structured algorithms that can guide PCPs toward more accurate assessment and management (11). Another review emphasized the importance of studying current diagnostic and management practices across healthcare systems and called for the development and testing of educational interventions for PCPs to improve patient outcomes (13).

A recent study from Switzerland identified key challenges in primary care management of dizziness, including limited consultation time, diagnostic complexity, and low referral rates to specialists despite the potentially serious underlying causes (12). These findings are consistent with a global study that revealed significant disparities in access to healthcare services for vestibular disorders across countries (14). In addition to diagnostic limitations, the economic burden of dizziness, through increased testing, repeated visits, and indirect costs such as lost productivity and work absenteeism, is substantial and often overlooked in policy discussions.

In Saudi Arabia, a recent population-based study reported a prevalence of dizziness exceeding 42%, a figure significantly higher than global averages and indicative of a potentially under-recognized public health issue (15). Gaps in structured training, limited referral networks, and inconsistent access to vestibular assessment tools may further contribute to underdiagnosis and mismanagement. Previous literature has not adequately assessed the diagnostic capacity, referral pathways, or continuing medical education of PCPs regarding vestibular disorders in this context. Given the increasing burden of non-communicable diseases and the Kingdom’s Vision 2030 (16) emphasis on strengthening primary care services, addressing this gap is both timely and essential.

Therefore, this study aims to assess the current status of dizziness and vertigo management in primary care settings in Saudi Arabia, with a focus on physicians’ diagnostic knowledge, satisfaction with available services, and their perspectives on needed improvements. Findings from this study are expected to inform targeted interventions, educational frameworks, and health policy decisions aimed at enhancing the quality of care for patients with vestibular disorders.

## 2 Materials and methods

### 2.1 Study design, participants and recruitment

A cross-sectional research design was employed to achieve the study’s objectives, targeting healthcare providers in primary care settings across Saudi Arabia. An online self-administered survey was developed specifically for this study (see Supplementary materials).

#### 2.1.1 Participant selection

- Inclusion criteria: (1) Currently practicing Primary Care Physicians, General Practitioners, or Family Medicine Physicians. (2) Actively practicing within the Kingdom of Saudi Arabia. (3) Working in the government or private sector.
- Exclusion criteria: (1) Medical students or interns not yet in residency. (2) Retired physicians. (3) Specialists in secondary or tertiary care (e.g., ENT surgeons, Neurologists) who are not the first point of contact for dizziness. (4) Practicing outside KSA.

#### 2.1.2 Sampling strategy

A convenience snowball sampling strategy was employed to recruit participants. The investigators initially distributed the electronic survey via professional social media platforms (LinkedIn and Twitter) and physician-specific WhatsApp groups. Participants were then encouraged to forward the survey to their peers within the primary care community. This method was chosen to maximize reach within the geographically dispersed population of Primary Care Physicians across the various regions of Saudi Arabia.

### 2.2 Survey

The survey consisted of 4 sections: (1) sociodemographic characteristics of the participants, (2) current practice regarding diagnosis/treatment of patients with dizziness, (3) knowledge about the assessment and treatment of dizziness, and (4) satisfaction with the provided service and suggestion for improvements.

The knowledge section consisted of 7 items, each rated on a 5-point Likert scale ranging from 1 (“Strongly disagree”) to 5 (“Strongly agree”). A composite knowledge score was calculated by summing the responses to all items, yielding a total score ranging from 7 to 35. Higher scores indicated greater knowledge regarding the diagnosis and treatment of dizziness and vertigo. Based on the total score, knowledge levels were categorized as follows: poor knowledge (score < 50%), moderate knowledge (50%–75%), and good knowledge (> 75%). While this tool provides a general overview of physician knowledge, it does not capture clinical decision-making and should be interpreted accordingly.

Satisfaction was assessed using a single-item question: “How satisfied are you with the results of the diagnostic workup initiated for patients presenting with acute or chronic/episodic dizziness as a main symptom?” Responses were rated on a 5-point Likert scale ranging from 1 (“Very dissatisfied”) to 5 (“Very satisfied”). For analytical purposes, satisfaction levels were reclassified into three categories: dissatisfied (scores 1–2), neutral (score 3), and satisfied (scores 4–5). While efficient, this single-item scale may not reflect the full complexity of satisfaction and should be considered a focused indicator. The estimated completion time of the survey was approximately 10 min.

The survey was piloted on a small group (10 physicians) to ensure face validity and clarity before full deployment.

### 2.3 Ethical consideration

Ethical approval for this study was obtained from the Institutional Review Board (IRB) at Princess Nourah bint Abdulrahman University, Riyadh, Saudi Arabia (IRB Approval Number: 23-0657). Participation in the study was voluntary, and all participants provided informed consent electronically before accessing and completing the survey.

### 2.4 Statistical analysis

Categorical variables were summarized using frequencies and percentages, while continuous variables were expressed as means and standard deviations (SD). Differences in knowledge and satisfaction scores across sociodemographic characteristics of PCPs were assessed using the Mann–Whitney U test and the Kruskal–Wallis H test.

Normality of distribution was evaluated using the Shapiro–Wilk and Kolmogorov–Smirnov tests, which indicated that both knowledge and satisfaction scores were not normally distributed. Therefore, non-parametric tests were applied for group comparisons. The correlation between knowledge and satisfaction scores was assessed using Spearman’s rank correlation coefficient.

A *p*-value of <0.05 was considered statistically significant. All statistical analyses were performed using IBM SPSS Statistics for Windows, version 26.0 (Armonk, NY: IBM Corp).

## 3 Results

A total of 186 PCPs participated in this study. The sociodemographic and practice-related characteristics of the respondents are summarized in Table 1. The majority of participants were younger than 30 years old (53.2%), and more than half were male (55.4%). Most respondents practiced in the Central Region of Saudi Arabia (71%), and over half had fewer than 5 years of clinical experience (57.5%).

**Table 1:** Sociodemographic and clinical practice characteristics of the participants (*n* = 186).

Study variables	*N* (%)
Age group	
<30 years	99 (53.2%)
30–40 years	67 (36.0%)
41–50 years	13 (07.0%)
51–60 years	02 (01.1%)
>60 years	05 (02.7%)
Gender	
Male	103 (55.4%)
Female	83 (44.6%)
Region of practice	
Eastern Region	17 (09.1%)
Central Region	132 (71.0%)
Northern Region	06 (03.2%)
Southern Region	06 (03.2%)
Western Region	25 (13.4%)
Years of professional experience	
<5 years	107 (57.5%)
5–10 years	44 (23.7%)
11–15 years	17 (09.1%)
16–20 years	07 (03.8%)
>20 years	11 (05.9%)
Duration spent with each patient	
<5 min	08 (04.3%)
5–10 min	72 (38.7%)
11–20 min	77 (41.4%)
21–30 min	20 (10.8%)
>30 min	09 (04.8%)
Practice settings	
Private	16 (08.6%)
Public	133 (71.5%)
Both	37 (19.9%)
Number of patients with a chief complaint of dizziness seen per month	
None	07 (03.8%)
1–2	32 (03.8%)
3–5	46 (24.7%)
>5	101 (54.3%)
Time spent with each patient with dizziness versus other patients	
Less time than other patients	16 (08.6%)
Same time as other patients	63 (33.9%)
More time than other patients	74 (39.8%)

Public hospitals were the most common workplace setting, reported by 71.5% of participants. More than half of the PCPs (54.3%) indicated that they encounter more than five patients per month presenting with dizziness. Regarding consultation duration, 41.4% of participants reported spending 11 to 20 min per patient. Notably, 39.8% stated that they typically spend more time evaluating patients with dizziness compared to other patients.

**Figure 1:**
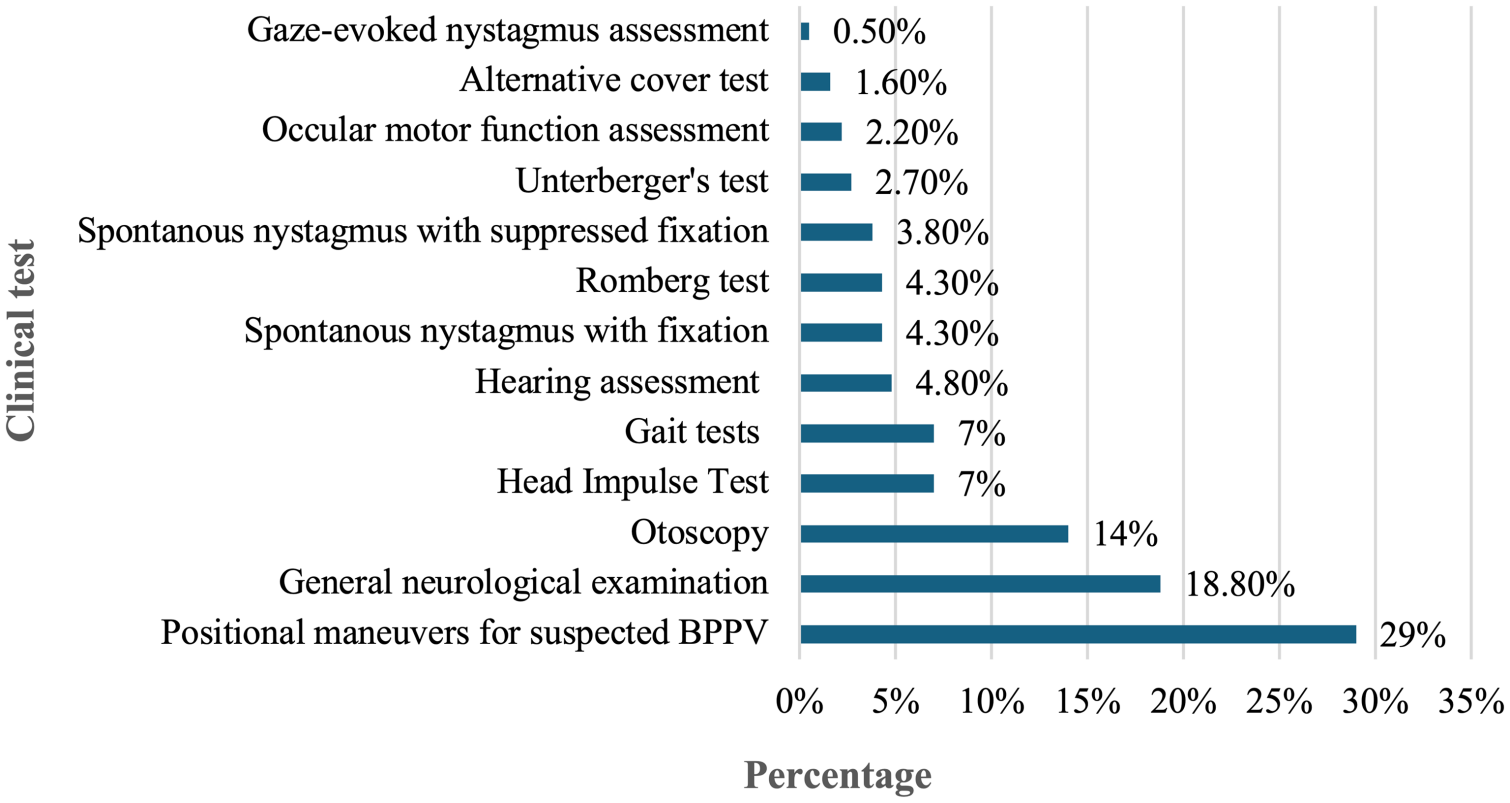
Participants’ ratings of the most important clinical tests to consider when diagnosing patients presenting with dizziness.

Figure 1 presents participants’ ratings of the most important diagnostic tests to consider when evaluating patients with dizziness. The five most highly rated procedures were: positional maneuvers for suspected benign paroxysmal positional vertigo (BPPV) (29%), general neurological examination (18.8%), otoscopy (14%), head impulse test (7%), and gait assessment (7%).

**Figure 2:**
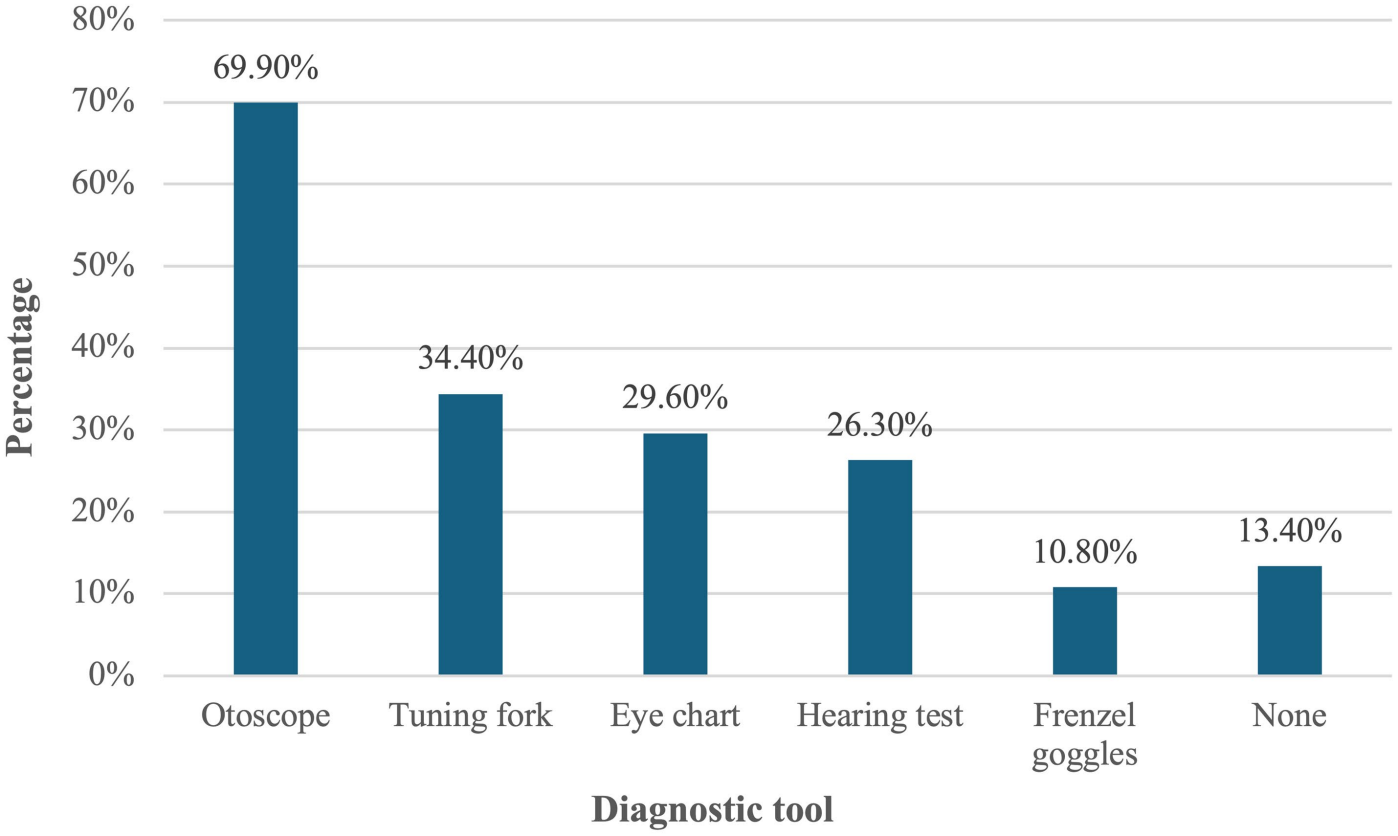
Types of diagnostic instruments available and used in clinical practice for the assessment of dizziness.

As illustrated in Figure 2, the most commonly available and used instruments for dizziness assessment in clinical practice were the otoscope (69.9%), tuning fork (34.4%), eye chart (29.6%), and hearing assessment tools (26.3%).

**Figure 3:**
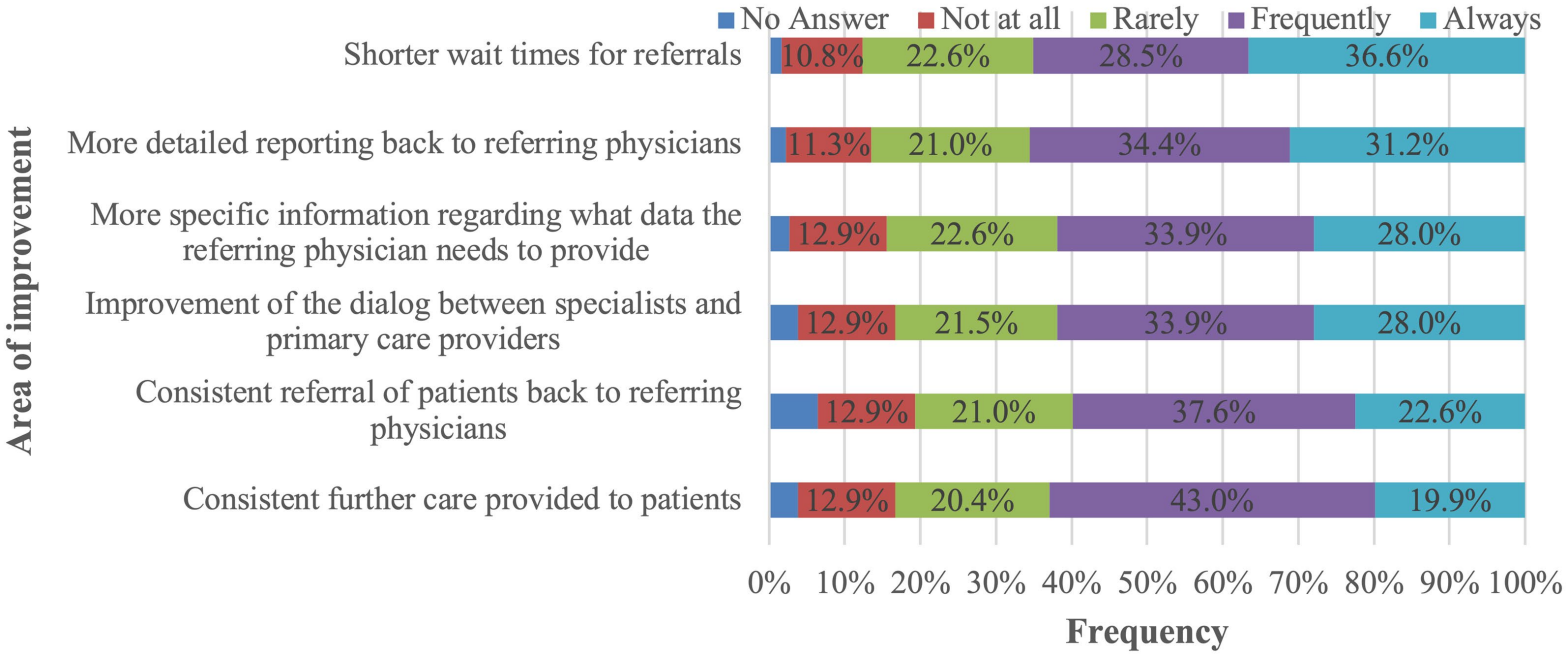
Frequency of perceived need for improvement in key areas to enhance the care of patients presenting with dizziness, as reported by participants.

Figure 3 displays the primary areas identified by PCPs as needing improvement to enhance the care of patients with dizziness. The top suggestions included reducing referral waiting times (36.6%), receiving more detailed specialist reports (31.2%), clearer guidance on required referral documentation (28.0%), and improved communication between PCPs and specialists (28.0%).

Table 2 summarizes the current practices of PCPs in managing patients presenting with dizziness. Approximately 27.4% of respondents reported referring between 10 and 30% of their dizzy patients to another specialist for further evaluation, with ear, nose, and throat (ENT) specialists being the most commonly selected referral option (60.2%).

**Table 2:** Current practices of participants in the assessment of patients presenting with dizziness (*n* = 186).

Statement	*N* (%)
Proportion of patients with dizziness referred to another specialist for further evaluation	
<10%	34 (18.3%)
10%–30%	51 (27.4%)
31%–50%	28 (15.1%)
51%–70%	17 (09.1%)
71%–90%	13 (07.0%)
>90%	04 (02.2%)
Not sure	39 (21.0%)
Specialists commonly referred to for dizziness	
ENT	112 (60.2%)
Neurology	45 (24.2%)
Emergency	21 (11.3%)
Interdisciplinary dizziness clinic	08 (04.3%)
Most common diagnoses for patients presenting with dizziness as a main symptom*	
BPPV (benign paroxysmal positional vertigo)	93 (50.0%)
Dizziness of unknown etiology	25 (13.4%)
Vestibular neuritis	14 (07.5%)
Somatoform dizziness (phobic vertigo)	12 (06.5%)
Dizziness/unsteady gait associated with polyneuropathy	11 (05.9%)
Multifactorial dizziness	08 (04.3%)
Ménière’s disease	08 (04.3%)
Cardiovascular causes	08 (04.3%)
Vestibular migraine	07 (03.8%)
Most common question to be considered when taking the history of patients presenting with dizziness*	
What type of dizziness occurred?	150 (80.6%)
Are there any ear disorders present (hearing loss, tinnitus, ear pain)?	119 (64.0%)
Is the dizziness triggered by certain movements?	116 (62.4%)
How long does an attack of dizziness last?	114 (61.3%)
Is the dizziness accompanied by nausea and vomiting?	108 (58.1%)
Are there any other symptoms accompanying the dizziness?	108 (58.1%)
Did the patient experience trauma beforehand (cranial, cervical spine)?	106 (57.0%)
Prior/current medication history?	105 (56.5%)
How often do attacks of dizziness occur?	95 (51.1%)
Is dizziness triggered in certain situations?	91 (48.9%)
Does the patient feel a tendency to fall in one direction?	84 (45.2%)
How intense is the dizziness?	79 (42.5%)
Symptoms in patients with dizziness that are considered for immediate referral for further investigation*	
Concomitant paralysis, dysarthria, dysesthesia, or vision problems	112 (60.2%)
Markedly unsteady gait	90 (48.4%)
Elevated blood pressure	89 (47.8%)
Nausea and vomiting	75 (40.3%)
Presence of nystagmus	67 (36.0%)
Accompanying unilateral, newly occurring hearing loss	63 (33.9%)
A tendency to fall when sitting unsupported or standing unassisted, requiring the patient to be caught.	58 (31.2%)
Isolated headaches	35 (18.8%)
Isolated tinnitus	22 (11.8%)

*Variable with multiple response answers.

The most frequently reported diagnosis was BPPV (50.0%), followed by dizziness of unknown etiology (13.4%). When obtaining a patient history, the most commonly asked question was, “Can you describe what your dizziness felt like?” (80.6%), followed by “Are there any ear disorders present?” (64.0%) and “Do certain movements trigger the dizziness?” (61.3%).

Regarding red flags for urgent referral, the most frequently selected symptoms were: concomitant neurological signs such as paralysis, dysarthria, dysesthesia, or vision problems (60.2%), markedly unsteady gait (48.4%), and elevated blood pressure (47.8%).

Table 3 presents participants’ knowledge regarding the assessment of patients with dizziness, rated on a 5-point Likert scale. The overall mean knowledge score was 20.9 (SD ± 3.99). Based on predefined cutoff values, 16.1% of participants demonstrated poor knowledge, 73.7% had moderate knowledge, and only 10.2% achieved a good knowledge level.

**Table 3:** Participants’ knowledge about the diagnosis of dizziness, their satisfaction with the provided services, and suggested methods to enhance knowledge in assessing and managing dizziness (*n* = 186).

Statement	Mean ± SD
Knowledge (scale 1–5)	
Antivertigo medications are the first-line treatment for patients with suspected BPPV	2.86 ± 0.91
Provocation maneuvers are the first-line treatment for patients with suspected BPPV	2.63 ± 0.96
Corticosteroids are recommended in the treatment of acute vestibular neuritis	3.02 ± 0.98
Referral to a specialist (e.g., ENT or neurology) is the only appropriate management for patients with acute vestibular neuritis	3.04 ± 1.02
Antivertigo medications are recommended for the long-term management of patients with chronic or episodic dizziness lasting more than 3 months	3.06 ± 0.98
Treatment with physical therapy (focused on balance training/gait training) are recommended for the long-term management of patients with chronic or episodic dizziness lasting more than 3 months	3.18 ± 0.97
Referral to Radiology for a cranial imaging is necessary for patients with acute vestibular neuritis	3.16 ± 0.99
Total knowledge score (mean ± SD)	20.9 ± 3.99
Level of knowledge	
Poor	*N* = 30 (16.1%)
Moderate	*N* = 137 (73.7%)
Good	*N* = 19 (10.2%)
Satisfaction (Scale 1–5)	Mean ± SD
How satisfied are you with the results of the diagnostic workup initiated for patients presenting with dizziness as a main symptom?	3.12 ± 0.98
Level of satisfaction	
Dissatisfied	*N* = 43 (23.1%)
Neutral	*N* = 85 (45.7%)
Satisfied	*N* = 58 (31.2%)
Suggested methods to improve knowledge of dizziness assessment and treatment*	*N* (%)
Hands-on courses/workshops	102 (54.8%)
Online lectures	85 (45.7%)
National practice recommendations and guidelines	84 (45.2%)
Smartphone apps to convey information	72 (38.7%)
Awareness campaigns	65 (34.9%)

*Variable with multiple response answers.

In terms of satisfaction with the current diagnostic services, the mean satisfaction score was 3.12 (SD ± 0.98). Overall, 23.1% of participants reported being dissatisfied, 45.7% were neutral, and 31.2% expressed satisfaction with the services provided for managing patients with dizziness.

Participants were also asked to suggest methods that could improve their knowledge and competency in assessing and managing dizziness, as detailed in Table 3.

Table 4 presents the associations between participants’ knowledge and satisfaction scores and their sociodemographic and clinical practice characteristics. Higher knowledge scores were significantly associated with more years of professional experience (*Z* = 2.016, *p* = 0.044), longer consultation times per patient (*Z* = 2.192, *p* = 0.028), and a greater number of dizziness-related cases seen per month (*Z* = 2.397, *p* = 0.017).

**Table 4:** Association between participants’ knowledge and satisfaction scores and their sociodemographic and clinical practice characteristics (*n* = 186).

Factor	Knowledge (maximum score: 35) Mean ± SD	Z/H-test; *p*-value	Satisfaction (maximum score: 5) Mean ± SD	Z/H-test; *p*-value
Age group^a^				
<30 years	20.5 ± 3.72	1.720; 0.085	3.07 ± 0.96	0.917; 0.359
≥30 years	21.4 ± 4.24		3.18 ± 0.99	
Gender^a^				
Male	20.7 ± 3.93	0.611; 0.541	3.19 ± 1.01	1.168; 0.243
Female	21.3 ± 4.06		3.04 ± 0.93	
Region of practice^a^				
Central Region	21.1 ± 3.82	0.508; 0.611	3.10 ± 0.96	0.476; 0.634
Other Regions	20.5 ± 4.36		3.19 ± 1.01	
Years of professional experience^a^				
<5 years	20.4 ± 3.68	2.016; 0.044**	3.00 ± 0.98	2.207; 0.027**
≥5 years	21.7 ± 4.28		3.29 ± 0.95	
Duration spent with each patient^a^				
≤10 min	21.6 ± 4.35	2.192; 0.028**	3.15 ± 1.02	0.552; 0.581
>10 min	20.5 ± 3.64		3.10 ± 0.95	
Practice settings^b^				
Private	20.1 ± 4.18	2.680; 0.262	2.69 ± 1.30	4.003; 0.135
Public	20.8 ± 4.09		3.12 ± 0.97	
Both	21.8 ± 3.46		3.32 ± 0.78	
Number of patients with a chief complaint of dizziness seen per month^a^				
≤5	20.3 ± 3.78	2.397; 0.017**	3.14 ± 1.00	0.350; 0.726
>5	21.5 ± 4.08		3.11 ± 0.96	
Time spent per dizziness patient versus other patients^b^				
Less time compared to other patients	20.3 ± 5.55	3.746; 0.154	3.00 ± 1.21	0.394; 0.821
Same time as other patients	21.1 ± 4.05		3.25 ± 0.99	
More time compared to other patients	21.8 ± 3.62		3.23 ± 0.88	

a Association assessed using Mann–Whitney *Z*-test.

b Association assessed using Kruskal–Wallis *H*-test.

**Significant at *p* < 0.05.

Satisfaction scores were also positively associated with years of professional experience (*Z* = 2.207, *p* = 0.027). However, no statistically significant differences in knowledge or satisfaction scores were found in relation to age, gender, region of practice, practice settings, or the time spent with patients presenting with dizziness (*p* > 0.05).

A statistically significant positive correlation was found between knowledge and satisfaction scores (rs = 0.495, *p* < 0.001), indicating that higher knowledge levels were moderately correlated with increased satisfaction among participants.

## 4 Discussion

This study assessed the current status of PCPs knowledge, diagnostic practices, satisfaction, and perceived needs related to the management of dizziness and vertigo in Saudi Arabia. The findings offer valuable insight into current practices and highlight gaps that must be addressed to improve care for patients with vestibular symptoms. Drawing upon established knowledge regarding the prevalence and impact of these conditions (3, 7–9, 15), the study sheds light on current practices, unveils areas for improvement, and explores the implications for stakeholders within the healthcare system.

While this study provides a broad overview of national trends, the participant pool was primarily composed of younger PCPs (<30 years old) with fewer than 5 years of clinical experience, and most were based in the Central Region, primarily in public hospitals. This demographic may not reflect more experienced physicians or those working in rural areas or private settings. Therefore, findings should be interpreted with caution and may not be generalizable to all regions or healthcare sectors. According to Gurajala (17), disparities between urban and rural regions remain a key challenge in developing an equitable healthcare system in Saudi Arabia.

Short consultation times were common, and only 39.8% of physicians reported spending additional time assessing dizzy patients, despite more than half managing over five cases monthly. While longer consultations are essential for accurate assessment, many PCPs reported allocating only standard time, possibly due to systemic time pressures. This aligns with global findings indicating that short consultations are common and often insufficient for managing complex conditions like dizziness (18). The implications of such short consultation lengths are concerning, as they are likely to adversely affect both patient healthcare and physician workload and stress. Patients may feel rushed and inadequately listened to, potentially leading to suboptimal care and dissatisfaction. Additionally, PCPs may experience increased stress and burnout due to the demands of managing patient concerns within constrained timeframes (18). This time constraint could contribute to the relatively high proportion of cases with “unknown etiology” (13.4%), exceeding prior estimates from a Saudi study (9.7%) (19). Such diagnostic uncertainty may stem not only from limited time but also from limited training, lack of access to appropriate tools, and variability in PCPs’ experience with vestibular disorders. Longer consultations not only provide opportunities for more comprehensive assessment and management of dizziness but also reduce the workload and stress on physicians, potentially leading to better patient outcomes.

Several factors likely contribute to limited consultation time, including lack of confidence in managing dizziness, high patient load, and the tendency to quickly refer to specialists (20–22). Furthermore, the perceived severity of symptoms may affect how much time PCPs invest in each case. For instance, non-urgent or mild symptoms may not prompt extended evaluation, even though they could be manifestations of significant vestibular disorders.

The availability of essential diagnostic tools was suboptimal. Otoscopes (69.9%), tuning forks (34.4%), and hearing tests (26.3%) were the most accessible. However, 13.4% of PCPs had no access to basic instruments, highlighting infrastructural limitations. To exemplify, the utilization of tuning forks was underscored regardless of its importance in cases of hearing loss (23) as it plays a crucial role in clinical assessment, particularly in identifying conditions such as cerumen impaction and tympanic membrane pathology. Cerumen impaction can lead to various distressing symptoms including hearing loss, ear fullness, itching, otalgia, tinnitus, cough, or, rarely, imbalance (24). The limited use of otoscopy might be attributed to the discomfort experienced by PCPs regarding head and neck examinations, particularly otoscopy, due to limited exposure during postgraduate medical education (25).

Moreover, the underutilization of tools like Frenzel goggles (10.8%) represents a significant concern. These simple bedside tools enable the detection of nystagmus (involuntary eye movements), a crucial indicator of vestibular dysfunction and various neurological conditions (26). Unlike advanced imaging, which provide static anatomical information, basic diagnostic tools offer a dynamic assessment of physiological function (27). Such tools can facilitate the observation of subtle physiological signs that might be missed on visual examination alone (28). This lack of access to essential diagnostic equipment can significantly hinder PCPs’ ability to make informed decisions regarding treatment plans, further diagnostic workups, specialist referrals, and even urgent care interventions (24). Additionally, the significance of diagnostic procedures such as positional maneuvers for BPPV and general neurological examination is underscored, particularly that BPPV is a prevalent condition (20).

Knowledge assessment revealed that while most participants had moderate knowledge (73.7%), 16.1% demonstrated poor understanding of dizziness evaluation aligning with existing research highlighting diagnostic challenges in primary care for dizziness (12, 24). This gap is significant given that inadequate knowledge can lead to delayed diagnoses, mismanagement, and inappropriate referrals (29). Similar gaps have been observed in emergency departments, where providers sometimes fail to recognize inner ear causes of dizziness (30, 31). Moreover, limited awareness of vestibular rehabilitation may further impact treatment decisions, as supported by studies showing low rates of physical therapy referral for dizzy patients (12, 32). However, while this suggests overall moderate competency, the lack of objective clinical assessments limits our ability to confirm true diagnostic proficiency.

Knowledge levels were significantly correlated with years of experience, time spent with patients, and the volume of dizziness cases seen, suggesting that real-world exposure contributes to improved clinical judgment. These findings support targeted educational interventions, such as workshops, national guidelines, and case-based learning, especially for less experienced PCPs (24, 33). This is further supported by participants’ own suggestions for such targeted interventions to enhance their knowledge (Table 3).

A positive association was observed between knowledge and satisfaction scores, suggesting that better-informed PCPs are more confident and content with their diagnostic approach. This finding aligns with previous studies. For instance, Mantokoudis et al. (24) reported that younger PCPs, with potentially less experience and knowledge, face greater difficulties reaching definitive diagnoses. Similarly, Young et al. (34) identified a correlation between knowledge gaps in PCPs and inappropriate Magnetic Resonance Imaging (MRI) referrals, while Zwergal et al. (12) found that PCPs with potentially lower knowledge levels in dizziness management were more likely to refer patients to specialists. These studies collectively suggest that diagnostic uncertainty driven by limited expertise may contribute to unnecessary specialist referrals in cases of vestibular disorders. Enhancing PCP knowledge through targeted training could therefore improve diagnostic confidence, reduce inappropriate referrals, and increase overall satisfaction with dizziness management.

The moderate satisfaction score (mean = 3.12) may reflect underlying frustration with system-level barriers, such as limited access to diagnostic tools, long referral waits times, and insufficient clinical training. However, the significant positive correlation with between satisfaction and knowledge (*r* = 0.495, *p* < 0.001) suggest that enhancing PCPs’ knowledge could meaningfully improve their confidence and satisfaction in managing dizziness. Strengthening physicians’ expertise in vestibular assessment and management has the potential to enhance diagnostic accuracy, improve patient outcomes, and support more efficient use of healthcare resources. It is important to note, however, that satisfaction in this study was measured using a single-item scale, which may not fully capture the multidimensional nature of PCPs’ experiences. Future studies could benefit from incorporating more comprehensive satisfaction measures to explore this relationship in greater depth. Given the observed training needs and system-level constraints, one promising strategy involves integrating audiologists into primary care teams. Audiologists bring expertise in vestibular assessment, triage, and rehabilitation, which can reduce referral delays and ease the burden on specialists (35, 36). Programs such as the Swansea Bay Audiology Pathway demonstrate that allied health-led models can effectively streamline care (37). Adapting such models to include vestibular concerns may enhance diagnostic accuracy, expedite care, and optimize resource utilization. This approach does not imply replacing PCPs in the assessment and management of dizziness. On the contrary, PCPs should continue to receive comprehensive training to ensure they can confidently and competently manage such cases. However, incorporating audiologists within primary care settings can serve as a valuable complement to enhance diagnostic accuracy, expedite care, and optimize resource utilization, particularly in settings with limited access to specialists.

### 4.1 Study limitations

This study has several limitations that should be acknowledged. First, the use of a self-reported electronic survey may introduce response bias, as participants may have overestimated or underestimated their knowledge and practices. Second, the cross-sectional design captures data at a single point in time, limiting the ability to assess causal relationships or changes in practice over time. Third, the sample was predominantly composed of younger physicians with fewer years of experience, most of whom practiced in the Central Region and within public hospitals. This limits the generalizability of findings to more experienced PCPs, those working in rural or underserved areas, and those in private practice settings. Regional disparities in healthcare access and resources, which may significantly influence diagnostic practices and satisfaction, were not explored in depth. Fourth, the knowledge assessment was based on a limited number of items and did not include case-based scenarios or objective evaluations, which may not fully reflect clinical competency. Similarly, satisfaction was measured using a single-item question, which may not capture the complexity of PCPs’ experiences and perceptions. Finally, while the study explored physicians’ suggestions for improving dizziness care, it did not include the perspectives of other stakeholders such as patients, audiologists, or specialists, which could provide a more comprehensive understanding of the care pathway and barriers to improvement.

### 4.2 Recommendations

- Prioritize hands-on workshops and e-learning modules focused on physical examination skills, especially for early-career PCPs.
- Implement mandatory, CME-accredited vestibular workshops to ensure primary care physicians achieve proficiency in standardized bedside vestibular assessment protocols.
- Develop and implement national clinical guidelines for dizziness management in primary care settings.
- Establish a national primary care protocol for managing benign vestibular conditions to achieve a significant reduction in unnecessary referrals to secondary and tertiary specialty clinics.
- Integrate audiologists into primary care teams to improve triage efficiency and reduce delays.
- Establish dedicated vestibular care pathways to optimize the use of healthcare resources and streamline patient care.

## 5 Conclusion

The findings of this study demonstrate a significant disparity between the perceived importance of dizziness management and the actual clinical proficiency of primary care physicians in Saudi Arabia. This disconnect suggests that in high-resource healthcare settings, clinicians may prioritize advanced neuroimaging over bedside diagnostic maneuvers, leading to a reliance on technology rather than physical examination.

The data of the study indicates that the underutilization of standardized vestibular assessments is not merely a lack of knowledge, but a systemic tendency to use primary care as a triage point for secondary referrals. Addressing this gap requires a shift in focus back toward strengthening core clinical skills, emphasizing that bedside vestibular evaluation is a cost-effective and highly sensitive primary care necessity. These assessments should be viewed as essential diagnostic tools for the first-contact clinician rather than specialized skills reserved exclusively for ENT or Neurology clinics.

## Data Availability

The raw data supporting the conclusions of this article will be made available by the authors, without undue reservation.
