# Supplementary material for: Knowledge, practices, and challenges in primary care management of dizziness and vertigo in Saudi Arabia
Source: Front Neurol. 2026 Mar 13;17:1793772. doi: 10.3389/fneur.2026.1793772 (PMC13021432; doi:10.3389/fneur.2026.1793772)
Supplement: Supplementary file 1 [file Data_Sheet_1.pdf]

Questionnaire

1. How old are you?

- <30 years
- 30–40 years
- 41–50 years
- 51-60 years
- >60 years

2. What is your gender?

- Male
- Female

3. How many years of professional experience do you have (since completing your medical training)?

- <5years
- 5-10 years
- 10-15 years
- 16-20 years
- > 20 years

4. On average, how much time do you spend with each patient?

- <5 minutes
- 5-10 minutes
- 11-20 minutes
- 21-30 minutes
- >30 minutes

5. Please select your practice setting.

- Private
- Public
- Both

6. Please select the geographic area of your practice in KSA.

- Eastern
- Western
- Central
- Northern
- Southern

7. How many patients with a chief complaint of dizziness do you see per month?

- o 1-2 patients
- o 3-5 patients
- o >5 patients
- o None

8. On average, how much time do you spend with each patient with dizziness versus other patients?

- o Less time than other patients
- o Same time as other patients
- o More time than other patients

9. What questions do you consider particularly important in diagnostic terms when taking the history of patients presenting with dizziness as a main symptom? Select all that apply

- o What type of dizziness occurred?
- o Are there any ear disorders present (hearing loss, tinnitus, ear pain)?
- o Is the dizziness triggered by certain movements?
- o How long does an attack of dizziness last?
- o Is the dizziness accompanied by nausea and vomiting?
- o Are there any other symptoms accompanying the dizziness?
- o Did the patient experience trauma beforehand (cranial, cervical spine)?
- o Prior/current medication history?
- o How often do attacks of dizziness occur?
- o Is dizziness triggered in certain situations?
- o Does the patient feel a tendency to fall in one direction?
- o How intense is the dizziness?

10. Which of the following tests you consider important when evaluating patients with dizziness? Select all that apply

- o Gait tests (tandem gait test, tandem gait test with eyes closed)
- o Romberg test
- o Unterberger's test
- o Assessment for spontaneous nystagmus with fixation
- o Assessment for spontaneous nystagmus with suppressed fixation (e.g. using Frenzel goggles)
- o Head impulse test

- Assessment for gaze evoked nystagmus
- Alternate cover test
- Hearing assessment
- Positional maneuvers for suspected benign paroxysmal positional vertigo (BPPV)
- General neurological examination
- Checking ocular motor function (evidence of ocular palsy?)
- Otoscopy

11. Which of the following testing instruments are available in your practice for you to use? Select all that apply

- Frenzel goggles
- Otoscope
- Eye chart
- Hearing test
- Tuning fork
- None

12. Approximately, what proportion of patients with dizziness do you refer to a specialist in another discipline for further diagnostic workup?

- <10%
- 10-30%
- 31-50%
- 51-70%
- 71-90%
- >90%
- I am not sure

13. To which specialists do you most often refer patients with dizziness?

- Neurology
- ENT
- Emergency
- Interdisciplinary dizziness clinic

14. What are your most common diagnoses for patients presenting with dizziness as a main symptom? Select all that apply

- BPPV (benign paroxysmal positional vertigo)
- Somatoform dizziness (phobic vertigo)

- Vestibular neuritis
- Dizziness/unsteady gait associated with polyneuropathy.
- Multifactorial dizziness
- Dizziness of unknown etiology
- Vestibular migraine
- Ménière’s disease
- Cardiovascular causes

15. In your opinion, which findings in patients with acute dizziness require immediate further investigation/diagnostic workup? Select all that apply

- Markedly unsteady gait
- Nausea and vomiting
- Concomitant paralysis, dysarthria, dysesthesia, or vision problems
- Presence of nystagmus
- Accompanying unilateral, newly occurring hearing loss
- A tendency to fall when sitting unsupported or standing unassisted, requiring the patient to be caught
- Isolated headaches
- Isolated tinnitus
- Elevated blood pressure

Please indicate your level of agreement with the following statements regarding the care of patients presenting with dizziness.

16. Antivertigo medications are the first-line treatment for patients with suspected BPPV.

- Strongly agree
- Agree
- Neutral
- Disagree
- Strongly disagree

17. Provocation maneuvers are the first-line treatment for patients with suspected BPPV.

- Strongly agree
- Agree
- Neutral
- Disagree
- Strongly disagree

18. Corticosteroids are recommended in the treatment of acute vestibular neuritis.

- Strongly agree
- Agree
- Neutral
- Disagree
- Strongly disagree

19. Referral to a specialist (e.g., ENT or neurology) is the only appropriate management for patients with acute vestibular neuritis.

- Strongly agree
- Agree
- Neutral
- Disagree
- Strongly disagree

20. Referral to Radiology for a cranial imaging is necessary for patients with acute vestibular neuritis.

- Strongly agree
- Agree
- Neutral
- Disagree
- Strongly disagree

21. Antivertigo medications are recommended for the long-term management of patients with chronic or episodic dizziness lasting more than 3 months.

- Strongly agree
- Agree
- Neutral
- Disagree
- Strongly disagree

22. Treatment with physical therapy (focused on balance training/gait training) are recommended for the long-term management of patients with chronic or episodic dizziness lasting more than 3 months.

- Strongly agree
- Agree
- Neutral
- Disagree
- Strongly disagree

23. How satisfied are you with the results of the diagnostic workup initiated for patients presenting with dizziness as a main symptom?

- Strongly dissatisfied
- Dissatisfied
- Neutral
- Satisfied
- Strongly satisfied

How often do you feel improvements are needed in the following areas to enhance the care of patients presenting with dizziness?

24. Improvement of the dialog between specialists and primary care providers.
- o Always
- o Frequently
- o Rarely
- o Not at all

25. Shorter wait times for referrals.
- o Always
- o Frequently
- o Rarely
- o Not at all

26. More specific information regarding what data the referring physician needs to provide when referring patients.
- o Always
- o Frequently
- o Rarely
- o Not at all

27. More detailed reporting back to referring physicians.
- o Always
- o Frequently
- o Rarely
- o Not at all

28. Consistent further care provided to patients.
- o Always
- o Frequently
- o Rarely
- o Not at all

29. Consistent referral of patients back to referring physicians.
- o Always
- o Frequently
- o Rarely
- o Not at all

30. Which of the following are appropriate steps to help you improve your familiarity with issues related to dizziness as a primary care provider? Select all that apply
- o Hands-on courses/workshops
- o Online lectures
- o National practice recommendations and guidelines

- Smartphone apps to convey information
- Awareness campaigns
